# Development of reverse-transcription loop-mediated isothermal amplification assays for point-of-care testing of human influenza virus subtypes H1N1 and H3N2

**DOI:** 10.5808/gi.22057

**Published:** 2022-12-30

**Authors:** Ji-Soo Kang, Mi-Ran Seo, Yeun-Jun Chung

**Affiliations:** 1 Department of Biomedicine and Health Sciences, Graduate School, The Catholic University of Korea, Seoul 06591, Korea; 2 Department of Microbiology, College of Medicine, The Catholic University of Korea, Seoul 06591, Korea; 3 ConnectaGen, Hanam 12918, Korea; 4 Precision Medicine Research Center, College of Medicine, The Catholic University of Korea, Seoul 06591, Korea; 5 Integrated Research Center for Genome Polymorphism, College of Medicine, The Catholic University of Korea, Seoul 06591, Korea

**Keywords:** influenza, influenza A virus, loop-mediated isothermal amplification, RT-LAMP

## Abstract

Influenza A virus (IAV) is the most widespread pathogen causing human respiratory infections. Although polymerase chain reaction (PCR)–based methods are currently the most commonly used tools for IAV detection, PCR is not ideal for point-of-care testing. In this study, we aimed to develop a more rapid and sensitive method than PCR-based tools to detect IAV using loop-mediated isothermal amplification (LAMP) technology. We designed reverse-transcriptional (RT)–LAMP primers targeting the hemagglutinin gene. RNAs from reference H1N1 and H3N2 showed specific RT-LAMP signals with the designed primers. We optimized the reaction conditions and developed universal reaction conditions for both LAMP assays. Under these conditions, the detection limit was 50 copies for both RT-LAMP assays. There was no non-specific signal to 19 non-IAV respiratory viruses, such as influenza B virus, coronaviruses, and respiratory syncytial viruses. Regarding the reaction time, a positive signal was detected within 25 min after starting the reaction. In conclusion, our RT-LAMP assay has high sensitivity and specificity for the detection of the H1 and H3 subtypes, making it suitable for point-of-care IAV testing.

## Introduction

Influenza viruses belong to the Orthomyxoviridae family and have a single-stranded negative-sense RNA genome consisting of eight gene segments, except for type C [1]. They encode 10 proteins: polymerase basic protein 1 (PB1), polymerase basic protein 2 (PB2), polymerase acidic protein, hemagglutinin (HA), neuraminidase (NA), nucleoprotein (NP), matrix 1 (M1), matrix 2 (M2), non-structural protein 1 (NS1), and non-structural protein 2 (NS2). Influenza viruses are classified into types A, B, and C according to the difference in antigenicity between the NP and M proteins. Influenza A virus (IAV) is sub-grouped according to the mutations in surface glycoprotein antigens: 18 HA subtypes (H1–H18) and 11 NA subtypes (N1–N11) [1-3].

Major outbreaks of influenza are always associated with influenza virus type A or B. In particular, IAV is the most widespread pathogen causing human respiratory infections. Human IAV (hIAV) has caused global pandemics, such as the H1N1 pandemic in 1918, the H2N2 pandemic in 1957, and the H3N2 pandemic in 2015, which can circulate in diverse animal hosts, including humans, birds, horses, dogs, and pigs [1,2,4,5]. The recent circulation of highly pathogenic avian H5N1 viruses in Asia since 2003 has caused human fatalities [6].

Due to the importance of the diagnosis of influenza subtypes, several influenza diagnosis methods have been developed [7-9]. Of the identification methods, quantitative reverse-transcriptional polymerase chain reaction methods are currently the most commonly used, with the advantage of sensitive detection of target viral RNA and determining the IAV subtypes; however, it takes at least several hours to obtain the result [7,10,11].

Loop-mediated isothermal amplification (LAMP) was developed by Notomi et al. (2000) [12], and this method can amplify DNA or RNA targets without thermal cycling. The LAMP reaction requires up to six target-specific primers. Unlike polymerase chain reaction (PCR), LAMP involves strand displacement using *Bst* DNA polymerase, which enables highly sensitive, quick, and efficient amplification under isothermal conditions [12]. In addition, LAMP reaction products can be read through turbidity or fluorescence [12-14], making the LAMP equipment simpler than the conventional PCR machines. These advantages have led to the further development of other molecular techniques, such as reverse-transcriptional loop-mediated isothermal amplification (RT-LAMP), by combining LAMP matrices with reverse transcription for the detection of infectious diseases caused by various pathogens [14]. The simplicity of RT-LAMP makes it ideal for field testing or diagnostics in point-of-care testing (POCT) settings with limited laboratory equipment, especially for RNA viruses [14]. The purpose of this study was to develop an RT-LAMP assay for the detection of hIAV H1N1 and H3N2. We also validated the sensitivity and specificity of this assay.

## Methods

### Primer design for the hIAV RT-LAMP

**Fig. 1.**
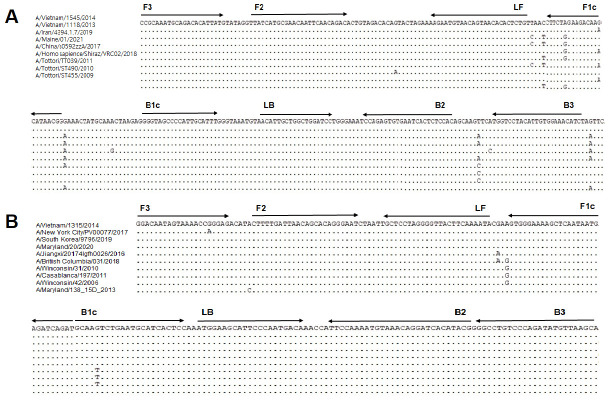
Nucleotide sequences used for designing the reverse-transcriptional loop-mediated isothermal amplification primers. Multiple sequence alignments of the H1-HA subtype (A) and H3-HA subtype (B) from BLAST are illustrated. Arrows indicate the positions of six primers (F3, B3, FIP [F2-F1c], BIP [B1c-B2], LF, and LB) aligned on the nucleotide sequence of the HA region. FIP, forward inner primer; BIP, backward inner primer; LF, loop forward primer; LB, loop backward primer; HA, hemagglutinin.

The HA gene sequences of hIAV subtypes H1N1 and H3N2 were obtained from GenBank (http://www.ncbi.nlm.nih.gov/genbank/) and aligned using BioEdit version 7.2 software (https://bioedit.software.informer.com/7.2/). The LAMP primers were designed using Primer Explorer version 5.0 (http://primerexplorer.jp/lampv5e/index.html) and manually curated. Each primer set contained two inner primers (forward inner primer [FIP] and backward inner primer [BIP]), two outer primers (forward outer primer [F3] and backward outer primer [B3]), and two loop primers that act to accelerate the reaction (loop forward primer [LF] and loop backward primer [LB]). All primers were then validated by the BLAST program. The information for the primer sets used in this study is presented in Fig. 1.

### RNA cloning and plasmid preparation

Influenza positive control strains, H1N1 and H3N2 virus RNAs, were obtained from the Korea Bank for Pathogenic Viruses. The HA gene of H1N1 and H3N2 was PCR-amplified and ligated into RBC T&A cloning vectors (RBC Bioscience, Taipei, Taiwan). After selecting the ligated clone, the extracted plasmid DNA was used as a template for RT-LAMP.

### RT-LAMP assay

RT-LAMP was performed as described in the previous studies with some modifications [12,13,15,16]. In brief, 20 μL of LAMP reaction mixture containing 2 μL of 10× isothermal amplification buffer (New England Biolabs, Ipswich, MA, USA), 1.5 mM concentrations of each deoxynucleotide triphosphate (New England Biolabs), 6 mM MgSO_4_ (New England Biolabs), 8 U of *Bst* 2.0 DNA polymerase (New England Biolabs), 7.5 U of WarmStart RTx Reverse Transcriptase (New England Biolabs), 1.25 μM SYTO9 (Thermo Fisher Scientific, Waltham, MA, USA), and 2 μL of template plasmid DNA of H1-HA or H3-HA was used. The composition of the RT-LAMP primer mix was 0.2 μM of the outer primers (F3 and B3), 1.6 μM of the inner primers (FIP and BIP), and 0.4 μM of the loop primers (LF and LB). The amplification reaction was performed at 60℃ for 45 min and terminated by heating at 80℃ for 3 min in a CFX96 Touch Real-Time PCR Detection System (Bio-Rad Laboratories, Hercules, CA, USA). The fluorescence curve was captured from the Bio-Rad CFX Manager graph.

### Optimization of hIAV RT-LAMP conditions

To optimize the amplification conditions, the LAMP reaction was performed at different conditions: reaction temperatures (60°C, 63°C, and 65℃), *Bst* DNA polymerase (4 U, 6 U, 8 U, and 10 U), dNTPs (1.0 mM, 1.2 mM, 1.4 mM, 1.6 mM, and 1.8 mM), and MgSO_4_ concentrations (4 mM, 6 mM, and 8 mM). Nuclease-free water was used as the negative control.

### Evaluation of specificity and sensitivity

To evaluate the specificity of our RT-LAMP assays, RT-LAMP reactions were performed with the RNA from 21 kinds of respiratory viruses (Table 1). To confirm the sensitivity of the RT-LAMP assay, plasmid DNA of H1-HA or H3-HA was serially diluted from 107 copies/μL to 25 copies/μL and applied in the RT-LAMP reaction. All amplification reactions were repeated three times.

## Results

### Design and validation of the hIAV RT-LAMP primer sets

For RT-LAMP, six primers were designed for the H1-HA and H3-HA genes, respectively: two inner primers (FIP and BIP), two outer primers (F3 and B3) and two loop primers (LF and LB) (Fig. 1). To validate whether the designed primers worked properly, the H1-HA and H3-HA RT-LAMP primer sets were applied to RNAs of reference H1N1 or H3N2 as described in previous studies (45 min at 60°C) [15,16], and it was confirmed that both RT-LAMP primer sets successfully amplified the reference H1N1 or H3N2 RNAs.

### Optimization of the hIAV RT-LAMP conditions

**Fig. 2.**
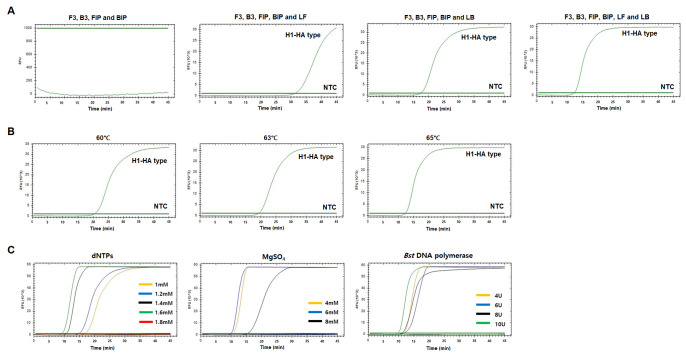
Optimization of human influenza A virus H1-HA reverse-transcriptional loop-mediated isothermal amplification (RT-LAMP). (A) RT-LAMP results with four different primer sets. (B) RT-LAMP results with three different temperatures. (C) RT-LAMP results with different concentrations of dNTPs, MgSO_4_, and *Bst* DNA polymerase. X-axis, time (min); Y-axis, relative fluorescence units (103). Nuclease-free water was used as a negative control (NTC).

**Fig. 3.**
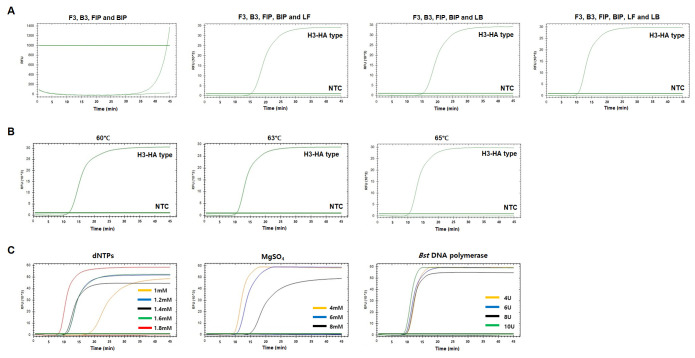
Optimization of human influenza A virus H3-HA reverse-transcriptional loop-mediated isothermal amplification (RT-LAMP). (A) RT-LAMP results with four different primer sets. (B) RT-LAMP results with three different temperatures. (C) RT-LAMP results with different concentrations of dNTPs, MgSO_4_, and *Bst* DNA polymerase. X-axis, time (min); Y-axis, relative fluorescence units (103). Nuclease-free water was used as a negative control (NTC).

We next optimized the RT-LAMP reaction conditions. We performed the RT-LAMP reactions with four different combinations of primer sets for H1-HA and H3-HA: primer set 1 contained two inner and two outer primers without loop primers, primer set 2 used the set 1 primers and LF, primer set 3 used the set 1 primers and LB, and primer set 4 contained the set 1 primers with both loop primers (LF and LB) (Figs. 2A and 3A). Of the four combinations, primer set 4 (all six primers) resulted in the fastest amplification in both H1-HA and H3-HA, and none of the negative controls showed positive signals (Figs. 2A and 3A). Therefore, we decided to include all six primers for both the H1-HA and H3-HA RT-LAMP assays. Under these conditions, we compared three different reaction temperatures (60°C, 63°C, and 65°C). Among them, 65°C showed the best amplification performance in H1-HA (Fig. 2B). However, in H3-HA, 63°C and 65°C showed almost identical performance (Fig. 3B). Therefore, we decided to use 65°C for both RT-LAMP assays. We also compared different concentrations of dNTPs (1.0 mM, 1.2 mM, 1.4 mM, 1.6 mM, and 1.8 mM), MgSO_4_ (4 mM, 6 mM, and 8 mM), and *Bst* DNA polymerase (4 U, 6 U, 8 U, and 10 U). As a result, the reaction conditions of 1.6 mM dNTPs, 6 mM MgSO_4_, and 10 U *Bst* DNA polymerase showed the fastest amplification without false-positive signals for H1-HA (Fig. 2C). For H3-HA, 1.8 mM dNTPs, 4 mM MgSO_4_, and 10 U *Bst* DNA polymerase were the best conditions (Fig. 3C).

### Sensitivity and specificity of the hIAV RT-LAMP assays

**Fig. 4.**
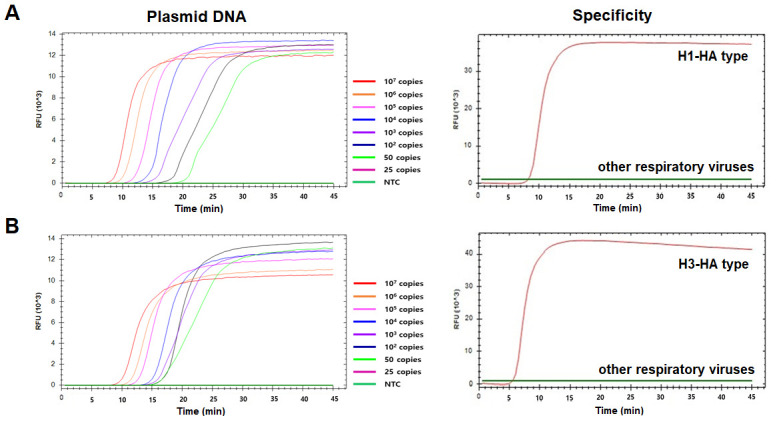
Sensitivity and specificity of H1-HA and H3-HA reverse-transcriptional loop-mediated isothermal amplification (RT-LAMP) assays. Serially diluted plasmid DNA of H1-HA (A) and H3-HA (B) subtypes was subjected to LAMP. Plasmid DNA containing H1-HA and H3-HA was diluted from 107 copies to 25 copies and subjected to RT-LAMP. To evaluate the specificity, 19 non-H1-HA and H3-HA respiratory viruses were tested. Nuclease-free water was used as a negative control (NTC).

To evaluate the detection sensitivity of hIAV RT-LAMP assays, we tested the limit of detection (LOD) of each assay by diluting target plasmid DNA from 107 copies/μL to 25 copies/μL. As a result, the LOD for H1-HA and H3-HA RT-LAMP was 50 copies of plasmid DNA per reaction (Fig. 4A and 4B). To test the specificity of the two RT-LAMP assays, we applied 21 kinds of respiratory viruses that can cause respiratory infections (Table 1). Only H1N1 and H3N2 showed a positive and specific amplification signal, respectively, and none of the other viruses, including influenza B, showed positive amplification (Fig. 4A and 4B).

## Discussion

Influenza virus is a pathogen that causes acute respiratory infections and can spread rapidly by airborne or direct person-to-person contact [17]. Therefore, rapid and accurate diagnostic tools for influenza infection are crucially important for patient management and the prevention of spread, especially for pandemic influenza [7,17,18]. Rapid influenza diagnostic tests (RIDTs) are rapid antigen-based immunoassays that are widely used in clinical settings because they are easy to use, the results can be obtained in a short time, and the test cost is low. However, due to the disadvantages of RIDTs, such as their low sensitivity and reliability, these tools are not suitable for early and sensitive detection [7,19]. reverse-transcriptional polymerase chain reaction (RT-PCR) is the most widely used method for influenza virus identification with good sensitivity and specificity. However, the complicated experimental procedures, including extracting viral RNA from a clinical sample, reverse transcription, and real-time amplification steps, take time and require expensive equipment, which is not ideal for an outpatient setting or POCT [7,11]. In this study, we aimed to develop a real-time RT-LAMP assay for the rapid and sensitive identification of major hIAVs suitable for POCT.

We designed LAMP primers targeting the HA segment of the hIAV H1-HA and H3-HA subtypes. There are 18 HA subtypes, each of which varies in amino acid sequences by at least 30%, and this variation plays an important role in determining host affinity [1-4]. Therefore, this method could be useful for the identification of hIAV subtypes. Through a comparison of different reaction temperatures and concentrations of key reagents (dNTPs and MgSO_4_), we defined a universal optimal reaction condition for both LAMP assays as follows: isothermal reaction at 65°C, 1.6 mM dNTPs, 6 mM MgSO_4_, 7.5 U of WarmStart RTx reverse transcriptase, and 10 U of *Bst* DNA polymerase in a reaction volume of 20 μL. Under these conditions, the LOD for the H1-HA and H3-HA subtypes was 50 copies (plasmid DNA) per reaction. Our results are consistent with previous studies reporting that RT-LAMP had higher sensitivity than PCR-based assays [20-24]. In particular, the reaction time of our RT-LAMP assay was less than 25 min, which was approximately four times quicker than that of the conventional RT-PCR assay. These results suggest that our RT-LAMP assays can identify IAV subtypes 20 times more sensitively and four times more quickly than conventional RT-PCR assays.

Considering the time for RNA extraction and preparation of reaction reagents (~30 min), physicians can receive the results within 1 hour after sampling from the patient, which is much shorter than PCR-based detection (~3 h) [9]. When we examined whether there were cross-reactions with other respiratory viruses (19 respiratory viruses), none of the assays showed cross-reactions with any of the respiratory viruses, suggesting high specificity of our RT-LAMP assays.

In conclusion, our hIAV H1-HA and H3-HA RT-LAMP assays could detect both targets with high sensitivity and specificity within 1 h. Considering the time and sensitivity/specificity, these assays would be a useful tool for detecting the hIAV H1-HA and H3-HA subtypes in the outpatient and POCT settings.

## Figures and Tables

**Table 1.** Respiratory viral strains used for testing cross-reactivity based on the RT-LAMP assay

No.	Organism	Source	RNA concentration (ng/μL)
1	Adenovirus 1	KBPV-VR-1D	174.2
2	Adenovirus 2	KBPV-VR-58D	146.7
3	Coronavirus OC43	KBPV-VR-8D	190.9
4	Coronavirus 229E	KBPV-VR-9D	95.7
5	Coronavirus NL63	KBPV-VR-88D	129.2
6	Parainfluenza virus 1	KBPV-VR-44D	121.6
7	Parainfluenza virus 2	KBPV-VR-66D	100.7
8	Parainfluenza virus 3	KBPV-VR-46D	117.6
9	Human rhinovirus 14	KBPV-VR-39D	113.9
10	Human rhinovirus 21	KBPV-VR-40D	126.6
11	Respiratory syncytial virus A	KBPV-VR-73	119.5
12	Respiratory syncytial virus B	KBPV-VR-48	128.4
13	Metapneumovirus	KBPV-VR-87D	106.1
14	Coxsackievirus A3	KBPV-VR-10D	108.3
15	Human Rotavirus A	KBPV-VR-47D	148.4
16	Enterovirus 71	KBPV-VR-56D	122.7
17	Measles virus	KBPV-VR-35D	112.6
18	Dengue virus 2	KBPV-VR-29D	128.4
19	Influenza B virus	KBPV-VR-94D	107.8
20	Influenza A virus (H1N1)	KBPV-VR-92D	333.4
21	Influenza A virus (H3N2)	KBPV-VR-93D	287.2

RT-LAMP, reverse-transcriptional loop-mediated isothermal amplification; KBPV, Korea Bank for Pathogenic Viruses.
